# The Structural and Functional Diversities of Bacteria Inhabiting Plant Woody Tissues and Their Interactions with Fungi

**DOI:** 10.3390/jof11090652

**Published:** 2025-09-03

**Authors:** Rana Haidar, Amira Yacoub, Ouiza Mesguida, Rémy Guyoneaud, Eléonore Attard, Patrice Rey

**Affiliations:** CNRS, IPREM, Université de Pau et des Pays de l’Adour, 64000 Pau, France; amira.yacoub@univ-pau.fr (A.Y.); ouiza.mesguida@univ-pau.fr (O.M.); remy.guyoneaud@univ-pau.fr (R.G.); eleonore.attard@univ-pau.fr (E.A.); patrice.rey@univ-pau.fr (P.R.)

**Keywords:** microbiome, bacterial communities, woody plants, grapevine, decaying wood, bcaterial/fungal interactions

## Abstract

In recent studies, the bacterial and fungal communities associated with plant wood have received considerable attention. Due to microorganisms’ vertical migration from roots to leaves, these communities provide critical links between the rhizosphere and phyllosphere microbiome. Recent investigations have shown that anatomical and chemical wood characteristics shape the microbiota inhabiting living or dead wood tissues, leading to variation in the observed decomposition of these materials. Despite the fact that bacteria have limited ability to degrade polymeric lignocelluloses compared to fungi, those inhabiting wood tissues have demonstrated a significant role in these habitats. Bacteria and fungi coexist in wood and form differing relationships with each other, with consequences for community structures that, in turn, impact plant health. The aim of this review is to present an overview of current insights regarding bacterial profiles and functions in lignocellulosic plants and their interaction with fungal communities colonising the same habitat. A better understanding of plant–bacteria–fungi interactions will allow for better exploitation of these tripartite interactions and possibly improve plant health.

## 1. Introduction

Plants constantly interact with microorganisms that colonise their tissues or the environment in which they are growing. What are the different types of microorganisms that colonise plants during the life cycle, and what are their different functions in plant health? These are central questions for today’s scientists to investigate. The plant-associated microbiome has several effects, including on seed germination and growth, nutrient supplementation, resistance to biotic and abiotic factors, and bioactive metabolite production [1]. In turn, the plant has a vital effect on its associated microorganisms, which are able to adapt to the different plant compartments and utilise plant-derived resources [2,3]. Aged woody plants are of interest because they represent the largest pool of above-ground terrestrial biomass, and their survival requires tolerance to a range of stresses such as freezing and drought. The interactions between woody plants and their associated microbiomes are complex, and the resulting networks play an essential role in plant growth and health [4,5,6]. Wood is essentially composed of cellulose, hemicellulose, and lignin. Each of these three polymers has a specific chemical structure and physical properties and is arranged in fibrils that form the cell wall matrix of each fibre. Due to its high lignin concentration, wood is a substrate that does not easily degrade. Despite lignin’s recalcitrance to degradation, various organisms (including fungi, bacteria, and insects) are able to degrade wood when the appropriate environmental conditions are met. Fungi have been described as pioneers in wood degradation due to their ability to produce numerous enzymes capable of efficiently degrading wood components (i.e., cellulose, hemicellulose, and lignin) [7,8]. The fungal secretion of various enzymes breaks down wood biopolymers, and microorganisms obtain functional nutrients that help them colonise wood tissues [9,10]. While white rot fungi degrade cellulose, hemicellulose, and lignin, brown rot fungi selectively degrade cellulose and hemicellulose. As bacteria’s contribution to wood decay is thought to be modest, more studies have focused on the fungal communities that colonise wood tissues [11,12,13]. However, there is increasing evidence that bacteria in wood tissues play a significant role in wood habitats, particularly regarding their chemical structure and physical properties in interacting with fungi, causing inhibited or enhanced development. Bacterial/fungal interactions happen naturally in wood as they co-occur in this environment; they interact both physically and functionally [14], resulting in mutual influence, as well as effects on the plant host. Recent research in this area has revealed the important diversity of bacterial communities in different woody species [15,16,17,18,19,20,21,22,23,24,25]. Understanding the relationships between fungi and bacteria inhabiting wood is of logical benefit for predicting physiological processes in woody species, such as decomposition or protection. In addition, the engineering of microbial communities in woody plants is of significant importance, especially for producing wood and timber and managing tree diseases.

Our objectives in this review are to discuss (i) the important bacterial community associated with the barks and various healthy or diseased woody tissues of different lignocellulosic species through community composition and function; (ii) the influence of abiotic, biotic (fungal) and physiological factors on wood bacterial communities that drive their structure and functions, and (iii) the complex interaction between wood-associated bacteria and fungi and their diverse functional roles in wood decay processes.

## 2. Bacteria’s Degradation of Wood Components

Since 1983, fungi have been considered to be primarily responsible for the biodegradation of wood, as they simultaneously break down its various polymers [26,27,28]. However, bacteria’s ability to decompose cell wood components, especially cellulose, was evidenced by Balows and Jennison (in 1949) [29] and then by Hulcher (in 1957) [30]. With regard to lignin, in 1962, Sorensen et al. were the first to show that several strains of bacteria isolated from soil and belonging to the genera *Pseudomonas* and *Flavobacterium* were able to degrade chemically prepared lignin. Various results showed that numerous bacterial strains from other genera, i.e., *Achromobacter*, *Aeromonas*, *Agrobacterium, Corynebacterium, Enterobacter, Klebsiella*, *Pseudomonas*, and *Streptomyces*, were able to degrade lignin [31,32,33,34]. These strains were isolated from a variety of sources, such as decaying wood, water, soil, and mud. Since 1990, there has been an increased interest in the role of bacteria in wood decay, combined with extensive research dedicated to the study of their ligninolytic activities [35,36,37]. Bacteria’s ability to decompose wood has been attributed to their versatile metabolism, producing cellulolytic, xylanolytic, pectinolytic, and ligninolytic enzymes [38,39,40]. The involvement of bacterial species in the transformation of lignin architecture was demonstrated in very diverse habitats, such as plants, soil, wastewater, and the termite gut [41,42]. Recent reviews have described the process of lignin depolymerisation caused by bacterial species and the different enzymes involved in breaking down lignin [37,38,43,44,45,46,47].

Regarding bacteria with cellulolytic and xylanolytic activities, numerous authors have demonstrated the abilities of diverse bacterial strains of different genera (e.g., *Bacillus* sp., *Paenibacillus* sp., *Clostridium* sp., *Cellulomonas* sp., *Ruminococcus* sp., *Erwinia* sp., *Streptomyces* sp., *Microbispora* sp., and *Fibrobacter* sp.) in hydrolising cellulose and hemicellulose [18,48,49,50,51].

**Figure 1:**
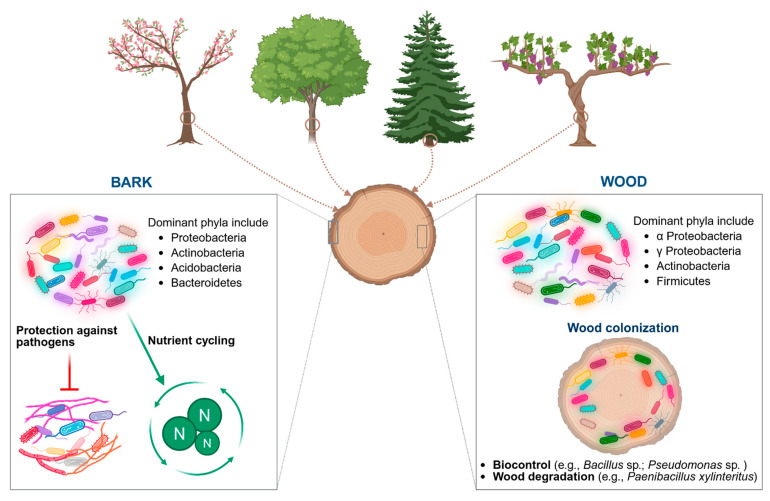
Bacterial diversity in plant bark and inner wood. Created in BioRender. Mesguida, O. (2025) https://BioRender.com/g0s8ixq (accessed on 3 July 2025).

Bacteria colonise different tree organs. The focus here is on bacterial diversity in the bark of woody plants, in inner wood, and particularly in vines (Figure 1).

## 3. Bacterial Diversity in Woody Plant Bark

Studying the microbiome diversity of bark is important because it represents the outermost part of the woody plant. The biological functions of bark are very diverse, including protection from environmental influences such as fire, pathogen attack, and herbivore damage, as well as water and photosynthate storage, sugar transport, and mechanical support for wound closure [52,53]. Although the microbiota of bark habitats has been less often explored than their phyllosphere or rhizosphere, recent research has confirmed bark as a reservoir of complex microbial diversity [54,55,56,57], with microbial communities distinct from other plant organs such as leaves, fruits, and roots [56,58,59]. For example, Jones et al. (2020) [60] reported that bacterial community composition was dependent on location; in bark, i.e., covered and exposed wood, the strongest differences were observed among four bacterial phyla, i.e., Chloroflexi, Firmicutes, Proteobacteria, and Spirochaetes, which contain the most important bacterial strains for wood decomposition [61,62]. A difference in mass loss between bark and wood was also observed. It has been suggested that the presence of bark slows wood decomposition and acts as a protective barrier against environmental exposure [60]. To investigate the effect of Norway spruce bark on the diversity of wood-inhabiting bacteria, Hagge et al. (2019) [63] either partly or completely debarked Norway spruce trees and compared their bacterial communities with those of control trees with intact bark. Higher bacterial richness was found in control trees with intact bark compared to partially or completely debarked trees. Thus, tree bark shapes the composition of wood-inhabiting bacterial communities, and bark’s moisture retention was suggested as the main reason for the higher bacterial richness in bark-covered trees. When Dreyling et al. (2022) [54] investigated the diversity of the bark-associated microbiome of beech (*Fagus sylvatica*) in Germany, they found highly diverse bacterial communities compared to the algae and fungi inhabiting the same bark tissues. As the genera *Methylocella* sp., *Acidiphilium*, and *Abditibacterium* are well adapted to wood conditions, they were dominant in the bark samples [54]. In a recent study, Dreyling et al. (2024) [64] studied the three main microbial groups (algal, fungal, and bacterial communities) of the bark surface of the dominant tree species—beech (*F. sylvatica*), pine (*Pinus sylvestris*), and spruce (*Picea abies*)—in three regions across Germany. The results showed that abiotic factors such as the relative humidity and light availability increased fungal diversity but decreased algal and bacterial diversity, which could lead to greater UV radiation sensitivity. Bacterial communities were primarily composed of taxa from the order Rhizobiales, followed by Acetobacterales, especially where pine was the dominant tree species [64]. Similarly, Hudson et al. (2023) [19] demonstrated that sampling site location, precipitation, height, isolation, and moisture were the main factors influencing bacterial diversity in the bark of red oak (*Quercus rubra* L.) at two sites in the mid-Atlantic region of the United States. The major phyla represented on the bark at both sites were Actinobacteria, Acidobacteria, Proteobacteria, and Bacteroidetes.

Other studies explored the bacterial community diversity in adjacent habitats of wood tissues, such as lichens, mosses, and bark. After comparing these, in Austria, Aschenbrenner et al. (2017) [65] showed that the microbial networks in lichen of *Lobaria pulmonaria* were less complex than those of mosses of *Pterigynandrum filiforme* and the bark of *Acer pseudoplatanus* (L.). While *Burkholderia*, *Pseudomonas*, and *Sphingomonas* were generally identified in each habitat, members of Rhizobiales were demonstrated to be particularly abundant in lichens. It has been suggested that the presence of *Sphingomonas* strains in all habitats is linked to their capacity to promote plant health by producing growth-promoting hormones.

In another *Acer* tree species, i.e., *Acer palmatum*, in Japan, Kobayashi and Aoyagi (2019) [66] reported that bacterial strains belonging to the phylum Actinobacteria were the most frequently isolated. The bark of this tree was suggested as a promising source of novel microorganisms, such as the newly identified bacterial strains IAP-33 and IAD21.

Vitulo et al. (2019) [55] reported on the importance of grapevine trunk bark as a rich bacterial diversity niche. They showed that grapevine stem bark harbours a greater diversity of epiphytic bacterial species than grape berries. Some bacterial families were specifically associated with bark samples (e.g., Acidobacteriaceae, Cytophagaceae, Rubrobacteraceae, and Sphingomonadaceae), while others were most important for characterising grape samples (e.g., Bacillaceae, Enterobacteriaceae, Oxalobacteraceae, and Paenibacillaceae). In terms of factors influencing bark bacterial communities, the growing region of the grapevine was identified as the most important, while growing practices, specifically conventional or biodynamic management, were described as the only factor influencing the grape microbiome [55].

In another study, Arrigoni et al. (2020) [57] reported that orchard location, seasonality, and disease management influence the composition of apple bark-associated bacterial communities, which may have adapted to disease management. In fact, potential plant pathogens increased, while some bacteria belonging to potential biocontrol genera decreased (e.g., *Aureobasidium*, *Filobasidium*, *Methylobacterium*, *Sphingomonas*, and *Sporobolomyces*), under low-input disease management. Regarding location, Aguirre-von-Wobeser et al. (2021) [59] reported that bacterial communities of avocado tree bark (*Persea americana*) had similar structures in two different orchards in Mexico, whereas these communities were distinct from those with rhizospheric soil, with only some taxa overlapping. The most abundant bacterial genera in these barks were *Sphingomonas* and *Methylobacterium*.

Bark age has been identified as a major driver of structural changes in bacterial communities. Arrigoni et al. (2018) [56] assessed the influence of tissue age on the composition of bacterial bark-associated communities of a scab-resistant apple cultivar. They observed that Nitrospirae, Planctomycetes, and Thermotogae were found only in young bark, while Chlamydiae and Chlorobi phyla were detected only in old bark. Similarly, on *Ginkgo biloba* trees, higher bacterial richness has been observed in old bark compared to young bark [58]. This study also reported greater bacterial diversity in bark samples compared to leaf samples. It has been suggested that bark acts as an environmental reservoir of plant microorganisms, with either positive or negative effects on plant health. In terms of positive effects, some bark-colonising bacteria are potential biocontrol agents, as shown by Dunlap et al. (2017) [67], who isolated, identified, and screened two bacterial strains from avocado bark in Florida (USA): *P. thiaminolyticus* and *P. apiarius*, which are antagonists against two avocado pathogens (*Raffaelea lauricola* and *Fusarium euwallaceae*). Interestingly, trunk bark can also harbor previously uncharacterised bacteria [68] and, in some cases, methanotrophic bacteria, such as *Methylomonas* [69] inhabiting the bark of *Melaleuca quinquenervia* in Australia. A new strain of *Pseudomonas* named *P. abieticivorans* sp. nov., which can grow on resin acids as the sole source of carbon, has been isolated from spruce bark [70].

## 4. Bacterial Diversity in Wood

Bacterial diversity in the wood tissues of plant species, whether in healthy or diseased states, has been evidenced by numerous authors [20,25,71,72,73]. Over the last decade, the function of these bacteria has received increasing attention [73,74,75,76]. The first studies on this topic reported that they are the first colonisers of wood, modifying its structure and permeability, thereby facilitating fungal infection [77,78].

Compared to fungi, bacteria have greater adaptability to a wide range of environmental conditions, as evidenced by their ability to tolerate wide ranges of temperature, oxygen, and pH. It has been reported that they are able to degrade cell wall components under extreme environmental conditions for basidiomycetes (e.g., moisture, reduced oxygen conditions, and toxic preservative and extractive levels) [41,79,80]. In aquatic environments, for example, the important role of bacteria in wood decay has been reported by Björdal and Dayton (2020) [81] and reviewed by Björdal (2012) [82]. The most important factors influencing the microbial degradation of wood in aquatic environments are likely to be temperature, salinity, oxygen, nutrients, and pH. Two types of bacteria—tunneling and erosion—and soft rot fungi (Ascomycetes and Fungi imperfecti) are the active microorganisms degrading wood in the marine ecological niche. In forests, on the dead wood of *F. sylvatica* logs, it has been reported that the bacterial community has better environmental fluctuation resistance than the fungal community [83,84]. Tláskal and Baldrian (2021) [85] reported that, on the dead wood of trees such as *F. sylvatica*, *P. abies*, and *A. alba*, the bacterial succession reflected their specific adaptations to increasing N content and C-containing compound fluctuations. However, compared to fungi, bacterial populations appeared to change more rapidly in response to climate change [86]. More recently, it has been shown that bacteria are heterogeneously distributed in dead wood compared to fungi, which are uniformly distributed [72].

### 4.1. Bacterial Diversity in Woody Grapevine Tissue

Bacteria have been particularly studied in grapevines. The influence of exogenous (e.g., climatic conditions, cultivation practices, geographical location) and endogenous factors (e.g., plant age and genotype) on the microbiota of grapevines has recently been reviewed by Bettenfeld et al. (2022) [73]. According to these authors, the plant microbiome is the most important determinant of plant health. Among these factors, the age of the grapevine influences bacterial composition, as shown by Andreolli et al. (2016) [87]. In this study, the diversity of endophytic bacteria colonising the stems of 3- and 15-year-old *Vitis vinifera* cv. Corvina vines in Italy was different. While actinobacteria and bacilli were frequently isolated from 3-year-old vines, alpha- and gamma-proteobacteria were more common in 15-year-old plants. Among these bacteria, they identified strains of the genus *Bacillus* that had growth-promoting properties and inhibited the growth of grapevine fungal pathogens such as *Botrytis cinerea*, *Neofusicoccum parvum*, *Phaeoacremonium aleophilum*, and *Phaemoniella*. *chlamydospore* [87].

Plant organs also influence bacterial colonisation. Aleynova et al. (2022) [88] analysed the biodiversity of endophytic bacteria in the shoots, leaves, berries, and seeds of *V. amurensis* Rupr. Most of these endophytic bacteria were found in stems and leaves, while grape seeds had the lowest bacterial biodiversity, with two genera, i.e., Amycolatopsis and Pseudoalteromonas, found only in this organ. On the other hand, Hamaoka et al. (2022) [89] studied the shoot xylems of different *V. vinifera* cultivars (i.e., Cabernet Sauvignon, Chardonnay, Koshu, and Pinot Noir) in Japan. They reported that the profiles of the bacterial communities were dependent on vineyard region, cultivar, and shoot growth stage. Actinobacteria, Bacteroidetes, Firmicutes (Bacilli and Clostridia), and Proteobacteria (Alphaproteobacteria and Gammaproteobacteria) were the genera identified in the shoot xylems of these cultivars [89]. In the past decade, several studies have focused on grapevine trunk diseases (GTDs), mainly Esca, Botryosphaeria, and Eutypa dieback, and the microbial communities that colonise the healthy and degraded wood of these diseased plants. The development of wood necrosis is a long process that takes place over years [90,91], and among the different necrotic wood tissues observed in GTD-affected plants, sectoral and central necrosis are common, as well as white rot, which is typical of Esca [92,93,94,95]. Wood-colonising bacteria have been studied in different grapevine cultivars sampled from vineyards in Argentina, Australia, Greece, France, and Tunisia. These bacteria have different functions that affect the health of the plants they colonise. The antagonistic activity of some of these indicates their biocontrol potential.

Regarding the microbiome of the *V. vinifera* cultivar, Malbec, with or without symptomatic ‘hoja de malvón’ foliage, a specific GTD of Argentina, Paolinelli et al. (2022) [96] analysed metatranscriptomic wood tissue. They showed that Actinobacteria, Bacteroidetes, and Proteobacteria were the predominant bacterial phyla. *Devosia* sp., a nitrogen-fixing bacterium, was among the well-represented genera in the grapevine wood bacterial community characterised in this study. The negative interaction between Propionibacteriales and several GTD pathogens (such as *N. parvum*, *Diplodia corticola*, and *Fomitiporia mediterranea*) suggests them as candidates for wood disease biocontrol. Bekris et al. (2021) [97] determined the wood bacterial communities of three major Greek cultivars (Agiorgitiko, Vidiano, and Xinomavro), examining samples both expressing GTD leaf symptoms and not. Proteobacteria dominated the wood bacterial communities, mainly α- and γ-Proteobacteria, followed by Actinobacteria and Bacteroidetes. The families Sphingomonadaceae and Bacillaceae were the most common. In asymptomatic plants, however, bacterial communities were dominated by *Bacillus* and *Streptomyces*. These bacteria showed a negative co-occurrence pattern with some GTD-relevant fungal genera (e.g., *Phaeomoniella*, *Phaeoacremonium*, and *Seimatosporium*), suggesting that interactions between microorganisms shape microbial communities. In another study, the international grapevine cultivar Cabernet Sauvignon was used by Bruez et al. (2015) [24] to characterise the bacterial communities colonising different wood tissues (necrotic or otherwise) of anatomical grapevine parts (trunk and cordon) with or without Esca leaf symptoms. Although specific complexes of bacterial communities colonised the wood tissues, the differences between them mainly depended on the anatomical part of the grapevine plant, whether cordon or trunk. In this study, the genus *Bacillus* was the most abundant in all wood tissue samples, regardless of plant pathogenic status. The co-inoculation of *N. parvum* (one of the GTD fungi) with 14 of the different bacterial species isolated in this study did not affect the extent of fungus-induced wood necroses [24]. Recently, Bruez et al. (2020) [25] studied the bacterial communities in non-necrotic wood tissues of young vines of the same cultivar (with or without Esca leaf symptoms). Meta-barcoding analysis revealed the dominance of a few abundant bacterial taxa corresponding to *Pantoea* sp., *Bacillus* sp., *Paenibacillus* sp., *Enterobacter* sp., and *Stenotrophomonas* sp. Interestingly, a few bacterial taxa (*Sphingomonas* sp. and *Mycobacterium* sp.) were the most abundant in diseased plants with more degraded wood. The microbial association was hypothesised to be related to disease onset and necessary for the development of degraded wood (white rot in the case of GTDs). Similarly, Niem et al. (2020) [98] studied the bacterial diversity in vine wood from two vineyards in Australia with and without external symptoms of GTDs. Their results showed that the abundance of *Pseudomonas* sp. in symptom-free vine tissues was higher than in symptomatic tissues. The dominant phyla were Proteobacteria, Actinobacteria, Bacteroidetes, Firmicutes, and Chloroflex. As they were the most abundant in healthy grapevine, the antagonistic activity of 10 *Pseudomonas* strains against 14 fungal species involved in GTDs was tested. The most efficient strains in inhibiting mycelial growth belonged to the *P. poae* group [98]. Cultivar is considered to be one of the most important factors influencing the grapevine bacterial microbiome. In Tunisia, *Pantoea*, *Pseudomonas*, *Curtobacterium*, and *Bacillus* were the most abundant cultivable strains isolated from necrotic and non-necrotic wood tissues of Muscat d’Italie cultivars showing symptoms of GTDs [99]. A strain of *B. subtilis* was able to significantly inhibit necrosis caused by *N. parvum* on grapevine stems of Muscat d’Italie plants under greenhouse conditions [99]. In another study on Sauvignon blanc cultivars, which also showed foliar symptoms, bacterial strains belonging to the families Pseudomonadaceae and Xanthomonadaceae were the most frequently isolated bacteria from the same types of wood tissues [49]. These authors demonstrated different bacterial effects in vitro against *F. mediterranea* (one of the main pathogens of GTD). While some strains were able to inhibit the mycelial development of *F. mediterranea* (e.g., *Pseudomonas* sp., *Stenotrophomonas* sp., *Novosphingobium* sp., and *Achromobacter* sp.), other strains did not display antagonistic activity (e.g., *Pseudoxanthomonas* sp., *Chryseobacterium* sp., and *Paenibacillus* sp.). In another grape variety, Sauvignon blanc, the same types of wood tissues in the canopy and trunk were sampled and analysed by Haidar et al. (2021) [49], who isolated 237 cultivable bacterial strains from Esca-affected grapevines. Most of these bacterial strains belonged to the families Pseudomonadaceae and Xanthomonadaceae. This study demonstrated the ability of fungi to degrade grapevine wood, which can be influenced by bacterial colonisation. While some of the bacterial strains were able to inhibit one of the major GTD fungi (*F. mediterranea*), other strains, especially a new species of *Paenibacillus* (named *P. xylinteritus*), were able to degrade cellulose and hemicellulose and promote the degradation of grapevine wood by this fungus [49,100].

### 4.2. Bacterial Diversity in Woody Tree Tissues

While microbial habitats such as the phyllosphere and rhizosphere have received considerable attention, a growing body of research indicates that the diverse microbial communities that colonise the wood tissues of living trees, and that appear to be adapted to their ecological niches, are likely to influence tree health. Many studies have compared microbial interactions, including those between pathogens and potential endophytic biocontrol agents, in healthy or diseased organs/tissues of trees when plant diseases occur. Depending on the tree species chosen and the experiments conducted, microbial diversity has been associated with disease in some way or another.

For example, Ren et al. (2019) [101] investigated the structure of bacterial communities in asymptomatic Norway spruce (*P. abies*) trees and in Heterobasidion-rotten symptomatic trees. This disease, triggered by the fungal pathogen *Heterobasidion annosum*, causes significant losses to forests in northern regions [102,103]. Under field conditions, Ren et al. (2019) [101] showed that, with the exception of needles, the pathogen did not induce shifts in the bacterial population of other plant organs, i.e., roots, bark, lower stem, and upper stem in symptomatic and asymptomatic Norway spruce trees. A chemical analysis of terpenoid compounds in the xylem and phloem vessels of spruce showed high concentrations of monoterpenes and sesquiterpenes in asymptomatic trees; however, no significant correlations were found between terpenoid profiles and bacterial community composition.

In an experiment with a relatively similar aim, Singh et al. (2019) [104] compared bacterial communities from the soil, rhizosphere, roots, and shoots of apple tree scions and rootstocks that did or did not show rapid apple decline. This disease is characterised by the collapse and decline of young apple trees, but its pathogen is still unknown. Bacterial communities in the soil and rhizosphere were significantly different from other plant tissues, but the abundance of bacterial classes did not differ in the shoot, soil, and rhizosphere of symptomatic and asymptomatic apple tree samples. Only one class of proteobacteria, alphaproteobacteria-rickettsiales, showed differential abundance in the roots of symptomatic and asymptomatic samples [104].

Greater microbial community differences between diseased and healthy trees have also been demonstrated in other experiments. For instance, Proença et al. (2017) [105] reported an increase in bacterial diversity with increased pinewood nematode *Bursaphelenchus xylophilus*-induced disease severity, with the highest diversity levels being observed in the most affected trees. A similar phenomenon was observed by Ren et al. (2021) [106] when they studied the bacterial communities in the twigs and leaves of Chinese chestnut trees infected (or not) with a Candidatus-phytoplasma; the asymptomatic samples had a higher bacterial diversity than the symptomatic equivalents. *Pseudomonas* and *Stenotrophomonas* were proposed to play major roles in asymptomatic leaf structures.

Some of these bacteria show potential biocontrol activities for plant protection. Liu et al. (2019) [107] showed that, among the gamma-proteobacteria, the most abundant in the stem of four Pinus species (*Pinus densiflora*, *P. koraiensis*, *P. rigida*, and *P. thunbergii*) in different sampling sites in Korea, two strains of *Escherichia coli* and one of *Serratia marcescens* showed nematocidal activity against the pine wood nematode *B. xylophilus*.

Differences in bacterial communities between soil and above-ground samples have been observed. An example of this was found by Izumi et al. (2008) [108], who investigated the diversity of cultivable endophytic bacteria colonising root, stem, and leaf tissues of Scots pine (*P. sylvestris* L.), silver birch (*Betula pendula* Roth), and rowan (*Sorbus aucuparia* L.) in Scotland. They showed that the bacterial communities associated with below-ground samples (roots and rhizosphere) were different from those associated with above-ground samples (leaves and stems) from the trees studied. Bahram et al. (2022) [109] came to a similar conclusion when comparing the bacterial structures associated with the different organs/compartments (leaves, branches, stems, sapwood, and roots) of two tree species (*Alnus incana* and *Betula pubescens*). Bacterial communities were significantly more distinct between above- and below-ground tree compartments, but not between hosts.

In some cases, this difference was related to bacterial function, as shown by Ren et al. (2019) [101]. They studied the endophytic bacterial community from different tissues (leaf, flower, fruit, stem, and root) of Jingbai pear trees in northern China. The high abundance of cyanobacteria in above-ground tree tissues (leaf, flower, fruit, and stem) was attributed to their photosynthetic profiles, suggesting their active role in the tissues studied.

## 5. Bacteria in Decaying Wood

A number of studies have focused on bacterial diversity and functions specifically in dead wood habitats. Zhang et al. (2008) [61] reported high bacterial diversity in decaying sapwood and heartwood from the conifer *Keteleeria evelyniana*, with higher bacterial richness in the latter than in the former. Chemical differences between these two types of wood, i.e., more lignin and less cellulose in the heartwood, could explain this observed difference. During the different phases of *Abies alba*, *F. sylvatica*, and *P. abies* wood decay in the south of the Czech Republic, Tláskal and Baldrian (2021) [85] showed that some of the dominant dead wood bacteria had high potential in terms of cellulose, chitin, hemicelluloses, and pectin utilisation.

Wood decomposition is a lengthy process involving biotic and abiotic factors, resulting in chemical and physical changes to the substrate [110]. Decaying wood contributes to ecosystem functions such as carbon sequestration, nutrient cycling, and forest biodiversity [111,112,113,114]. As wood density decreased during *P. abies* decay, an increase in the abundance and richness of bacteria was observed compared to archaea, suggesting that decayed wood is more hospitable to prokaryotes [22]. In decaying wood in forests, bacterial communities showed high diversity [21,61] and, together with fungi, they are key components in the decomposition of dead wood and transforming its complex organic compounds into simpler substances, such as nutrients, which are released into the wood and soil [12,85]. However, compared to fungi, the relative abundance fluctuation of bacteria across different decay classes was less obvious, as shown by Deng et al. (2022) [115] on eucalyptus stump substrate.

### 5.1. Bacteria Vary During Wood Decay Phases

Between the early and late decomposition stages, bacterial community profiles are different. According to Peng et al. (2020) [116], the diversity and distribution of the bacterial population in *Betula platyphylla* wood in China were influenced by changing wood properties during decomposition. For example, the genus *Methylacidiphilum* was mainly found in the later stages of decomposition when the wood was more decayed. Wood nitrogen content was a major factor influencing bacterial community composition, and alphaproteobacteria, which include several nitrogen-fixing bacterial taxa such as Rhizobiales, colonised *P. abies* wood during the early stages of decomposition [22]. However, mycophagous bacteria such as Burkholderiales, Granulicella, and Luteibacter tended to be more abundant in the later stages of decomposition when the fungal biomass in dead wood increased.

The major impact of certain environmental factors, such as decomposition time, pH, and water content, on bacterial community composition has also been reported [23]. Pastorelli et al. (2020) [117] assessed the composition of microbial communities associated with different stages of downy birch decomposition under a boreal climate. The abundance of the studied microbial groups (fungi, bacteria, actinobacteria, and archaea) increased with advancing decay. The succession of microbial taxa able to utilise available biopolymers probably explains this microbial abundance. A quite distinct bacterial population was observed in each dead wood decay stage, suggesting a successional pathway of diverse bacterial species during this process.

The abiotic factors influencing the bacteria inhabiting decaying wood include tree species, wood density, C:N ratio, C:N:P stoichiometry, pH, temperature, and moisture content [8,18,22,23,73,115]. Sample moisture content also had an effect on the bacterial richness of decaying wood [116]. In contrast to fungi, bacterial abundance was positively correlated with wood moisture. While bacteria dominated the microbial communities in the late stages of wood decay, fungi and actinobacteria were involved in the early stages of wood colonisation [117]. Hoppe et al. (2016) [8] compared the microbial communities of decaying *P. abies* and *F. sylvatica* logs and showed that Rhizobiales bacteria, known for their ability to fix nitrogen, were more abundant in the intermediate and advanced stages of decay [8].

### 5.2. Influence of Wood Decay on Soil

Dead wood and forest soils are examples of forest habitats that directly influence each other. For example, soils are thought to be an important source of microbial inoculum for dead wood [118,119,120]. Błońska et al. (2024) [121] showed that beech wood decay affected complex soil properties such as pH, N and C concentrations, and enzymatic activity. They also reported that the number of bacterial and fungal taxa was highest in the soil under the influence of decaying wood compared to the control. This could be due to the specific microenvironments created by decaying wood structures in the soil, which change at different stages of decomposition and influence microbial communities. Sun et al. (2014) [122] demonstrated that two different types of forest soil—mineral and pure peat—affected the microorganisms living in Norway spruce (*P. abies*). They placed autoclaved wood cubes in forest soil for five months, and although the composition of bacteria remained stable over the incubation period, the forest soil types significantly affected the community structures of wood-inhabiting bacteria. Other studies showed that the bacterial composition changes during the dead wood decomposition process as the number of bacterial successional taxa increases, resulting in a bacterial community more similar to that found in soil. Tláskal et al. (2017) [23] followed the development of bacterial communities in the dead wood of *A. alba*, *F. sylvatica*, and *P. abies* in a temperate natural forest in the Czech Republic, finding that bacterial taxa typical of forest soils (e.g., Bradyrhizobium, Rhodoplanes, and Steroidobacter) were more abundant in late decay.

## 6. Bacterial–Fungal Associations in Wood

The diversity and structure of fungal and bacterial communities associated with wood have been studied. Haq et al. (2022) [123] quantified the bacterial communities associated with *Fomes fomentarius* (white rot) and *Fomitopsis betulina* (brown rot) on birch (*Betula papyrifera*), showing that the fungal mechanisms of wood decay appear to be associated with different bacterial community compositions. Firmicutes were more abundant in *F. fomentarius* samples, whereas Acidobacteria and Proteobacteria were significantly more abundant in *F. betulina* samples. This was related to the fact that *F. fomentarius* degrades more lignin compared to *F. betulina*, which preferentially degrades holocellulose.

**Figure 2:**
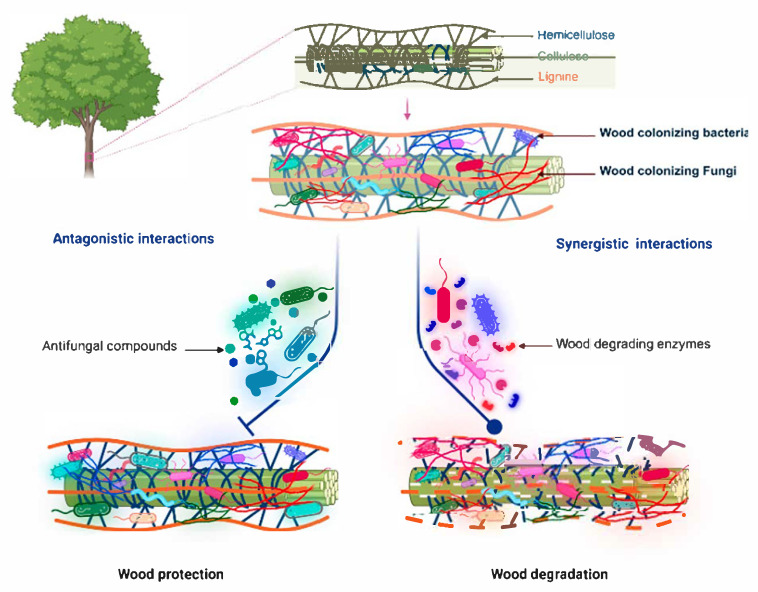
Functional interactions between the bacteria and fungi colonising plant wood. Created in BioRender. Mesguida, O. (2025) https://BioRender.com/ijxfm3l (accessed on 3 July 2025).

In wood tissues, bacteria and fungi are involved in various relationships ranging from antagonism to mutualism, which affect plant health [62,124,125] (Figure 2). It has been suggested that fungal communities may influence bacteria in dead wood, as fungi have primary access to carbon resources [126]. Fungi can also create a microenvironment with a lower pH and more reactive oxygen species, which could negatively affect the function and colonisation of bacterial communities [21,62,127].

These relationships between microorganisms also have implications for nutrition. For example, some studies have reported on bacteria’s ability to provide nitrogen to fungi in decomposing wood via nitrogen fixation [127,128,129,130]. Sometimes, bacteria feed on dead fungal biomass using various enzymes such as chitinase [131]. However, there are also examples of synergistic activity between fungi and bacteria in wood tissue, which are discussed in the next section.

### 6.1. Bacterial Synergistic Activity in Woody Plants

To address the issue of fungal rot’s influence on the bacterial community in decaying wood, several authors have employed a microcosm approach using wood sawdust. Hervé et al. (2014) [124] used beech sawdust and showed that the fungal pathogen *Phanerochaete chrysosporium* strongly influenced the diversity and structure of beech-associated bacteria (*F. sylvatica* L.). They also suggested that members of the genus *Burkholderia* were associated with this fungal pathogen, while, in another study, this research group reported that wood degradation was more important when *P. chrysosporium* was associated with the bacterial community isolated from the mycosphere [125]. The presence of *P. chrysosporium* resulted in the selection of cellulose- and hemicellulose-degrading bacteria, which may have contributed to the wood degradation process [125]. In grapevine, Haidar et al. (2021) [49] demonstrated the ability of many bacterial strains isolated from grapevine wood to degrade cellulose and hemicellulose in vitro. Using a grapevine sawdust microcosm approach, these authors also suggested that there was a synergistic relationship between the white rot fungus, *F. mediterranea*, and a new species of *Paenibacillus*, *P. xylinteritus* [100], which promoted grapevine wood structure degradation. In another recent study, Haidar et al. (2024) [132] showed that *F. mediterranea*’s ability to degrade wood was enhanced following the co-inoculation of sawdust from the cultivar Ugni blanc with two xylanolytic and cellulolytic strains of *Paenibacillus* (i.e., *Paenibacillus* sp. strain S231-2 and *P. amylolyticus* strain S293). The authors hypothesised that this bacterial–fungal cooperation is relatively common and may explain the high susceptibility of this cultivar to GTD. In another study of grapevine, strong synergistic effects were observed between the bacteria *B. pumilus* and/or *Xanthomonas* sp. and another fungus involved in GTDs, *N. parvum*, which aggravated disease symptoms in grapevine plants and facilitated the pathogenic process of *N. parvum* [133]. In addition to these fungal pathogens of wood, the complementary roles of fungi and bacteria inhabiting woody tissues have been demonstrated by Tláskal et al. (2021) [134], who showed that they facilitate the decomposition of dead wood in temperate forests. In the latter case, wood decomposition is the result of the specific roles of bacteria and fungi in C and N cycling. Cooperation between fungi and bacteria was not always observed; for example, the bacterial colonisation of fungally colonised beech (*F. sylvatica*) was delayed by decomposer fungi such as *Hypholoma fasciculare*, *Trametes versicolor*, or *Vuilleminia comedens*. This could be due to fungal selection pressure on the bacterial community, particularly on the Burkholderiales in the study by Christofides et al. (2019) [135].

### 6.2. Antagonistic Bacteria of Fungal Pathogens in the Woody Plant Tissues

The ability of bacteria to inhibit pathogenic fungi in woody plants has been well documented in the literature [136,137], such as in Mesguida et al.’s review (2023) [138] of grapevine trunk diseases. Reports have focused on trees used in industry for their wood, e.g., pine and poplar, or for the fruits they produce, e.g., avocado, cacao, citrus, grapevine, olive, and pistachio.

For pine, the *B. pumilus* strain HR10 has been described as an efficient biocontrol bacterium against Sphaeropsis shoot blight [139], preventing the invasion of pathogen hyphae into needles through the bacterial colonisation of pine tissues and surrounding the pathogen mycelium. On *P. densiflora* wood blocks, a strain of *Streptomyces* sp. showed strong inhibitory activity against the tested pathogens, especially *Gloeophyllum trabeum* [140]. Other bacterial strains of the genus *Bacillus* and *Burkholderia*, isolated from healthy stem tissue of *Pinus taeda*, produced metabolites with high inhibitory activity against *F. circinatum*, the pitch canker fungus [141]. For poplar, Ren et al. (2013) [142] selected a bacterial strain, *B. pumilus* JK-SX001, which was able to colonise poplar tissues and promote plant development, in addition to its biocontrol function against poplar canker fungi such as *Cytospora chrysosperma*, *Fusicoccum aesculi*, and *Phomopsis macrospora*. On the other hand, Li et al. (2020) [143] used a *S. sioyaensis* strain isolated from poplar plantation soils as a biocontrol agent against poplar canker caused by *Valsa sordida*. This *S. sioyaensis* strain inhibited the pathogen, either directly by suppressing mycelial growth and spore germination, or indirectly by increasing cell membrane permeability and disrupting the pathogen’s metabolic pathways. Another bacterial strain, *B. velezensis* EB14, was used by Naik et al. (2021) [137] as a biocontrol agent against stem leaf spot and poplar canker caused by *Sphaerulina musiva*. The production of cyclic lipopeptides with antifungal activity (e.g., iturin and fengycin) has been suggested as one mode of action of this bacterium.

For fruit trees, especially apple, *B. amyloliquefaciens* strain GB1 isolated from cucumber stems and *Saccharothrix yanglingensis* isolated from cucumber roots were effective against apple valsa canker caused by *Valsa mali* [143,144]. The inhibition of fungal conidia germination and fungal hyphae growth was observed with *B. amyloliquefaciens* GB1, which was also able to colonise phloem and xylem tissues around wounds on apple twigs and form biofilms [143]. The *S. yanglingensis* strain colonised apple tissues and secreted antibiotic substances that inhibited pathogen infection [145]. Against *Neonectria ditissima*, another fungal pathogen that causes European apple canker, Liu (2019) [146] reported that many apple endophytic bacterial species isolated from apple shoots had biocontrol activity. For example, two *Pseudomonas* strains protected wounds from *N. ditissima*, while *Bacillus* strains inhibited *N. ditissima* mycelial growth in vitro by producing diffusible antifungal metabolites. In avocado, three *Paenibacillus* species and one *Bacillus* species isolated from the bark of Florida avocado trees were effective against two pathogens: *F. euwallaceae* and *R. lauricola* [67]. The efficacy of *P. thiaminolyticus* and *P. apiarius* against a phytopathogen was reported for the first time in this study. According to Melnick et al. (2011) [147], several endophytic bacterial strains in cocoa trees show potential antagonistic activity against *Theobroma cacao* pathogens. However, when discussing their in vitro and detached leaf screenings, the authors concluded that their tests either excluded bacterial strains that could reduce plant disease or selected bacteria that might increase disease, showing the limitations of their experiments.

For citrus, Daungfu et al. (2019) [148] reported that many *Bacillus* strains have potential as biocontrol agents for citrus canker. They showed that three endophytic bacteria isolated from healthy citrus plants (*B. amyloliquefaciens* LE109, *B. subtilis* LE24, and *B. tequilensis* PO80) proliferated within host plant tissues, formed endospores, and displayed antagonistic activity against *Xanthomonas citri*. When leaves were inoculated with cell suspensions of these bacterial strains, citrus canker was completely controlled.

In olive (*Olea europaea* L.), *P. fluorescens* PICF7 has been reported as an effective biological control agent against *Verticillium dahliae*-induced Verticillium wilt. This PICF7 strain exhibits swimming motility, which contributes to its ability to colonise olive roots [149]. The antagonistic activity of endophytic *B. velezensis* strains isolated from olive trees against *Xylella fastidiosa* ST53 was demonstrated by Zicca et al. (2020) [150], with culture filtrates of these strains able to produce inhibitory activity against ST53.

For wild pistachio trees, Etminani and Harighi (2018) [151] reported the plant growth-promoting ability and biocontrol activity of several bacterial strains of *Bacillus*, *Pantoea*, *Pseudomonas*, *Serratia*, and *Stenotrophomonas* against bacterial pathogens, namely *P. syringae* pv. *syringae* Pss20 and *P. tolaasii* Pt18. Auxin and gibberellin, two plant growth hormones, were produced at different levels by each of these protective strains, and one strain was able to produce a siderophore and solubilise phosphate. Decaying wood was also reported as an ecologically rich niche and a source of microorganisms of potential biotechnological interest [152]. These authors demonstrated the ability of strains of isolated species (e.g., *Penicillium expansum*, *S. fimicarius*, *S. niveus*) to produce enzymes (including cellulases, endoglucanases, and feruloyl esterases) with the capacity to degrade different molecules present in lignocellulosic biomass.

## 7. Conclusions

Compared to the rhizosphere and phyllosphere microbiome, wood-associated microorganisms have received less attention as an ecological habitat. Bacterial community structures in wood have been shown to be influenced by environmental factors; by tree species, health, and decay stage; and by fungal decay type. Fungi are major contributors to wood decay, resulting in physical structural changes. Despite the recent increase in the number of research projects investigating the abundance and function of bacteria in bark and wood tissues, little information is available on their relationship with wood-decaying fungi and their dynamics during wood decomposition. By understanding the structure and function of bacterial communities, we can better understand their interactions not only with fungi but also with plants and, consequently, modify these interactions to improve plant health. Further research is also needed to investigate the effect of other factors, such as geometry, moisture, and temperature, on microbial interactions in different woody plant species and their effect on wood decay. In addition, as the bioconversion of lignocellulosic biomass is considered an alternative source of bioenergy, harnessing bacterial abilities in this regard is a promising avenue for research.
